# Killing Two Birds With One Stone – Strain Engineering Facilitates the Development of a Unique Rhamnolipid Production Process

**DOI:** 10.3389/fbioe.2020.00899

**Published:** 2020-08-07

**Authors:** Isabel Bator, Tobias Karmainski, Till Tiso, Lars M. Blank

**Affiliations:** ^1^iAMB – Institute of Applied Microbiology, ABBt – Aachen Biology and Biotechnology, RWTH Aachen University, Aachen, Germany; ^2^Bioeconomy Science Center (BioSC), Forschungszentrum Jülich, Jülich, Germany

**Keywords:** *Pseudomonas*, metabolic engineering, synthetic biology, adaptive laboratory evolution, ethanol, rhamnolipid, fermentation, biosurfactants

## Abstract

High-titer biosurfactant production in aerated fermenters using hydrophilic substrates is often hampered by excessive foaming. Ethanol has been shown to efficiently destabilize foam of rhamnolipids, a popular group of biosurfactants. To exploit this feature, we used ethanol as carbon source and defoamer, without introducing novel challenges for rhamnolipid purification. In detail, we engineered the non-pathogenic *Pseudomonas putida* KT2440 for heterologous rhamnolipid production from ethanol. To obtain a strain with high growth rate on ethanol as sole carbon source at elevated ethanol concentrations, adaptive laboratory evolution (ALE) was performed. Genome re-sequencing allowed to allocate the phenotypic changes to emerged mutations. Several genes were affected and differentially expressed including alcohol and aldehyde dehydrogenases, potentially contributing to the increased growth rate on ethanol of 0.51 h^–1^ after ALE. Further, mutations in genes were found, which possibly led to increased ethanol tolerance. The engineered rhamnolipid producer was used in a fed-batch fermentation with automated ethanol addition over 23 h, which resulted in a 3-(3-hydroxyalkanoyloxy)alkanoates and mono-rhamnolipids concentration of about 5 g L^–1^. The ethanol concomitantly served as carbon source and defoamer with the advantage of increased rhamnolipid and biomass production. In summary, we present a unique combination of strain and process engineering that facilitated the development of a stable fed-batch fermentation for rhamnolipid production, circumventing mechanical or chemical foam disruption.

## Introduction

Biosurfactants are surface-active molecules with high industrial potential due to their wide range of applications. They can be used in, e.g., bioremediation, enhanced oil recovery, plant protection, cosmetics, and medicine (Lang and Wullbrandt, 1999; Maier and Soberón-Chávez, 2000; Banat et al., 2010; Silva et al., 2014). In contrast to fossil-based surfactants, biosurfactants can be produced from renewable resources, exhibiting often a low ecotoxicological potential (Henkel et al., 2012; Johann et al., 2016). While the number of biosurfactants described is still increasing, they usually belong to the classes of glycolipids, phospholipids, lipopeptides, or polymeric biosurfactants (Desai and Banat, 1997).

The glycolipid rhamnolipid was first reported in 1949 by Jarvis and Johnson (Jarvis and Johnson, 1949). The production of rhamnolipids is mainly a trait of the bacterial genera *Pseudomonas* and *Burkholderia* (Germer et al., 2020), where the opportunistic pathogen *Pseudomonas aeruginosa* is one of the most reported species. Rhamnolipids consist of a hydrophobic moiety, one or two β-hydroxy fatty acids, and a hydrophilic moiety, one or two rhamnose molecules, which are linked through a β-glycosidic bond to the fatty acid chains (Edwards and Hayashi, 1965; Syldatk et al., 1985). More than 20 different congeners are produced by *P. aeruginosa*, while the most abundant congener contains a C_10_-C_10_ β-hydroxy fatty acid (Déziel et al., 1999, 2000). For the synthesis, two pathways for the precursor molecules are needed, which exist in *Pseudomonas* species. The synthesis of dTDP-L-rhamnose is catalyzed by five enzymes, a phosphoglucomutase and four enzymes of the *rml*-operon, starting from D-glucose-6-phosphate (Rahim et al., 2000). The β-hydroxyacyl-ACP is synthesized in the fatty acid *de novo* synthesis. The genes *rhlA* and *rhlB*, which are organized in an operon, are involved in mono-rhamnolipid synthesis (Ochsner et al., 1994; Déziel et al., 2003). The acyltransferase RhlA links two β-hydroxyacyl-ACP molecules to a dimer called 3-(3-hydroxyalkanoyloxy)alkanoate (HAA). Then, the rhamnosyltransferase RhlB adds a rhamnose to the HAA using dTDP-L-rhamnose as substrate. If one more rhamnose is added by the rhamnosyltransferase RhlC a di-rhamnolipid is produced (Rahim et al., 2001). RhlC is encoded by *rhlC*, which is located in another genomic region of *P. aeruginosa*.

The production of rhamnolipids with *P. aeruginosa*, although in industry established, encounters some challenges. First, the sophisticated regulation of the rhamnolipid synthesis genes by quorum sensing (Soberón-Chávez et al., 2005; Dusane et al., 2010), which complicates fermentation procedures. Second, the highest rhamnolipid production is achieved using plant oils as substrates (Sim et al., 1997), which increases the effort and costs in downstream processing. Third, the before mentioned pathogenicity is critical for the permission of production processes and the resulting products. Thus, rhamnolipid production with a non-pathogenic host where the production is independent from host intrinsic regulation using renewable hydrophilic substrates is much aimed for. Many studies report the heterologous production of rhamnolipids in different organisms, e.g., *Escherichia coli*, *P. fluorescens*, and *P. putida* KT2440 (Ochsner et al., 1995; Wang et al., 2007; Wittgens et al., 2011). The latter species is a well-established chassis in industrial biotechnology and is known for its versatile metabolism and high redox cofactor regeneration rate (Ramos et al., 1995; Nelson et al., 2002; Blank et al., 2010; Tiso et al., 2014).

Apart from the general microbial fermentation challenges, biosurfactant fermentations potentially have extensive foaming and high purification costs as further challenges. Excessive foaming is especially a challenge in aerated and agitated biosurfactant cultivations using hydrophilic substrates. The foaming facilitates biomass and product loss from the liquid phase. The product can be enriched in the foam phase by more than 20 times (Beuker et al., 2014, 2016b). Losing the whole-cell catalyst has a negative impact on volumetric production rate (Davis et al., 2001). While foaming is a true challenge, several strategies to control or prevent foaming exist. Antifoaming agents (or antifoamers) can be used, with the potential drawbacks of lowering the oxygen transfer rate, increasing the cost as they are expensive, and complicate downstream processing due to emulsification (Vardar-Sukan, 1998; Beuker et al., 2014). Another alternative is the use of mechanical foam breakers, which destroy the foam by shear stress (Junker, 2007). These elements have an additional energy demand and need maintenance, thus increasing also process costs. Further alternatives exist, e.g., adsorption of cell-containing foam, bubble-free membrane reactors, and foam fractionation (Winterburn et al., 2011; Beuker et al., 2014; Anic et al., 2017). However, all control or prevention methods have their limits, and usually add complexity to the fermentation. Hence, the development of alternative strategies to simplify the process, e.g., by controlling foaming, is a crucial step in process design and should be addressed in an early stage. Avoiding, e.g., antifoamers reduces elaborative downstream processing and hence costs.

In times of massive production of greenhouse gases, the usage of alternative feedstocks is highly recommended. The overall goal is to establish a circular bioeconomy in which also lignocellulosic biomass and side- and waste streams are used as energy and carbon source. Several studies deal with the production of rhamnolipids from alternative resources, e.g., butane, agro-industrial waste, glycerol, organic acids derived from bio-oil, and xylose (Gehring et al., 2016; Gudiña et al., 2016; Tiso et al., 2017; Arnold et al., 2019; Bator et al., 2020). In the 1990s, ethanol was already considered as an alternative carbon source for rhamnolipid production (Matsufuji et al., 1997). Bioethanol is an interesting substrate because it can be produced from biomass constituents like sugars or sugar polymers (e.g., starch) (Wendisch et al., 2016). But also attempts to use lignocellulosic sugars for bioethanol were made in the last decades, and various cellulosic ethanol plants are in operation (Balan et al., 2013; Duwe et al., 2019; Rosales-Calderon and Arantes, 2019). Further, synthesis gas (syngas) fermentations can be used to produce ethanol from greenhouse gases (Bengelsdorf and Dürre, 2017). A syngas-based industrial-scale ethanol production facility using *Clostridium autoethanogenum* as biocatalyst was brought into operation in 2018 (LanzaTech, 2018). Hence, CO and CO_2_ can directly be used as carbon sources for sustainable ethanol production. The produced ethanol can be used as biofuel, as platform chemical (e.g., ethylene or ethylene glycol synthesis) or as a carbon source for the production of valuable products as proposed here for the synthesis of rhamnolipids.

In this work, we report heterologous rhamnolipid production with engineered *P. putida* KT2440 from ethanol. We used adaptive laboratory evolution (ALE) to improve growth on and tolerance toward ethanol and identified the basis for adaptation by genome re-sequencing. An evolved mutant was characterized regarding growth and then engineered for mono-rhamnolipid production. Due to the improved growth parameters of the strain in the presence of ethanol, we were able to develop a unique rhamnolipid production process where ethanol takes over two functions – being the carbon source and the defoaming agent.

## Materials and Methods

### Strains and Cultivation Conditions

All bacterial strains used in this study are listed in Table 1. *E. coli* strains, *P. putida* KT2440 (DSM6125, ATCC47054), and engineered *P. putida* strains were routinely cultivated in lysogeny broth (LB) medium (10 g L^–1^ peptone, 5 g L^–1^ yeast extract, and 10 g L^–1^ NaCl). If solid medium was needed, 2% (w/v) agar was added. Cultivation of *P. putida* strains was performed at 30°C and *E. coli* strains were cultivated at 37°C. To avoid the loss of plasmids, 50 μg mL^–1^ kanamycin or 30 μg mL^–1^ gentamycin were added to the medium. Quantitative microbiology experiments were performed using M9 minimal medium with a final composition (per L) of 8.5 g Na_2_HPO_4_⋅2H_2_O, 3 g KH_2_PO_4_, 0.5 g NaCl, 1 g NH_4_Cl, 2 mM MgSO_4_, 4.87 mg FeSO_4_⋅7H_2_O, 4.12 mg CaCl_2_⋅2H_2_O, 1.5 mg MnCl_2_⋅4H_2_O, 1.87 mg ZnSO_4_⋅7H_2_O, 0.3 mg H_3_BO_3_, 0.25 mg Na_2_MoO_4_⋅2H_2_O, 0.15 mg CuCl_2_⋅2H_2_O, 0.84 mg Na_2_EDTA⋅2H_2_O (Sambrook and Russell, 2001), and 10 g glucose for pre-cultures or different concentrations of glucose and ethanol for main cultures as indicated. Liquid cultivations were performed in 500 mL shake flasks with 10% filling volume at 300 rpm and in 24-deep well plates (SystemDuetz; Enzyscreen B.V., Heemstede, Netherlands) with 1 mL filling volume at 300 rpm and a throw of 50 mm. A Growth Profiler 960 (Enzyscreen B.V., Heemstede, Netherlands) was used for online analysis of growth without sampling. 96-well plates with 200 μL filling volume at 225 rpm were incubated and the density was determined by image analysis.

**TABLE 1:** Bacterial strains used in this study.

Strains and plasmids	Characteristics	References
***E. coli***		
PIR2	F^–^, Δ*lac169*, *rpoS*(*Am*), *robA1*, *creC510*, *hsdR514*, *endA*, *recA1 uidA*(Δ*Mlu*I):*pir*; host for *oriV*(R6K) vectors	Thermo Fisher Scientific
HB101 pRK2013	Sm^*R*^, *hsdR*-*M*^+^, *proA2*, *leuB6*, *thi-1*, *recA*; harboring plasmid pRK2013	Ditta et al., 1980
DH5α pSW-2	*supE44*, Δ*lacU169* (Φ*80lacZ*ΔM15), *hsdR17* (r_*K*_^–^ m_*K*_^+^), *recA1*, *endA1*, *thi-1*, *gyrA96*, *relA1*; harboring plasmid pSW-2 encoding I-*Sce*I nuclease, tool for genomic deletion	Martínez-García and de Lorenzo, 2011
PIR2 pEMG-*fleQ*	PIR2 harboring plasmid pEMG-*fleQ*	this study
DH5αλ*pir* pTNS1	λ*pir* lysogen of DH5α; harboring plasmid pTNS1	Choi et al., 2005
DH5αλ*pir* pSK02	DH5αλ*pir* harboring Tn7 delivery vector pSK02 for chromosomal integration; containing *rhlAB* genes from *P. aeruginosa* PA01	Bator et al., 2020
***P. putida***		
KT2440	wild type	Bagdasarian et al., 1981
KT2440 E1	Isolate of *P. putida* KT2440 after 23rd transfer from laboratory evolution on ethanol	This study
KT2440 E1.1	Isolate of *P. putida* KT2440 after 33rd transfer from laboratory evolution on ethanol	This study
KT2440 Δ*fleQ*	Δ*fleQ*	This study
KT2440 SK4	wild type with *att*Tn7:P_*ffg*_*-rhlAB*	Tiso et al., 2020
KT2440 E1.1_RL	KT2440 E1.1 with *att*Tn7:P_*ffg*_*-rhlAB*	This study

### Adaptive Laboratory Evolution

For adaptation to ethanol, two consecutive ALE experiments were carried out. Each experiment was performed in two biological replicates: *P. putida* KT2440 was grown in M9 minimal medium containing 2 g L^–1^ glucose and 4% (v/v) ethanol. The optical density (OD_600_) was measured and fresh medium was inoculated with a starting OD_600_ of 0.1 daily. After 30 days (23 transfers), the medium composition was changed to 4% (v/v) ethanol. ALE was carried on for twelve more days (10 transfers). To obtain single isolates, the inhomogeneous culture was plated on LB-agar. Subsequently, single colonies were tested for adaptation to ethanol in a 96-well plate in a Growth Profiler 960 (Enzyscreen B.V., Heemstede, Netherlands).

### DNA Techniques

Genome re-sequencing of the wild type, *P. putida* KT2440 E1, and *P. putida* KT2440 E1.1 was performed to identify mutations obtained by ALE on ethanol. Therefore, genomic DNA was isolated with the High Pure PCR Template Preparation Kit (Roche Holding, Basel, Switzerland). The sequencing was done by Eurofins Genomics (Ebersberg, Germany) using Illumina technology as paired-end reads of 150 base pairs. Single nucleotide polymorphisms (SNPs) and insertions or deletions (InDels), which were analyzed by GATK’s Haplotype Caller (McKenna et al., 2010; Depristo et al., 2011), were visualized with the Integrative Genomics Viewer (Robinson et al., 2011). The sequences of the wild type, *P. putida* KT2440 E1, and *P. putida* KT2440 E1.1 were deposited in the Sequence Read Archive with the accession number (PRJNA642834).

Expression levels of genes were determined by quantitative real-time PCR (qRT-PCR). RNA was extracted from the wild type, *P. putida* KT2440 E1, and *P. putida* KT2440 E1.1 when growing in M9 minimal medium containing 0.96% (v/v) ethanol. The medium was inoculated at a starting OD_600_ of 0.1 and the cells were cultivated until they reached an OD_600_ of 1.1 mL of the cultures was centrifuged at 16,000 × *g* for 2 min, the supernatant was discarded, and the cells were resuspended in 1 mL RNA*later* Stabilization Solution (Thermo Fisher Scientific, Waltham, MA, United States). All further steps for RNA extraction were performed using the Monarch Total RNA Miniprep Kit (New England Biolabs, Ipswich, MA, United States) following the manual. The RNA concentration was measured with the NanoDrop One (Thermo Fisher Scientific, Waltham, MA, United States) at 260 nm. All samples were adjusted to a concentration of 6 ng μL^–1^ in a total volume of 40 μL. After RNA isolation, an additional DNase digestion was performed for 1 h at 37°C using 5 μL DNase I and 5 μL DNase I reaction buffer (New England Biolabs, Ipswich, MA, United States). The DNase I was inactivated at 75°C for 10 min. 4 μL of the RNA template were used for cDNA synthesis, which was performed using the ProtoScript II First Strand cDNA Synthesis Kit (New England Biolabs, Ipswich, MA, United States) following the manual. Subsequently, PCR was performed in white 96-well plates using the Universal qPCR Master Mix (New England Biolabs, Ipswich, MA, United States) in a CFX Connect Real-Time PCR Detection System (Bio-Rad Laboratories, Hercules, CA, United States). For qRT-PCR, specific oligonucleotides with annealing temperatures of 60°C were designed using the Primer3 software^1^. Standard curves for all primer pairs were performed by using two-fold dilution series of genomic DNA of the wild type with seven data points. Afterward, the primer efficiencies were determined and reached values between 2 and 2.13. Nuclease-free water for every primer pair and DNase treated RNA templates were used as negative controls. Each cDNA sample was diluted 1:10 and analyzed as technical triplicate. Data analysis was performed using the Bio-Rad CFX Manager Software. The resulting Ct values were used to calculate the gene expression level via the 2^Δ*Ct*^ method by normalizing to the house keeping gene *rpoB*.

For the deletion of the *fleQ* gene (PP_4373) the I-SceI-based system developed by Martínez-García and de Lorenzo (2011) was used. Briefly, 500 bp upstream and downstream flanking regions were amplified from the genomic DNA of *P. putida* KT2440 with the Q5 High-Fidelity polymerase (using primers IB-278, IB-279, IB-280, and IB-281) and cloned into the non-replicative pEMG vector by Gibson Assembly (Gibson et al., 2009). All used primers with their nucleotide sequences are listed in the Supplementary Material (Supplementary Table 1). The resulting plasmid pEMG-*fleQ* was transferred into chemically competent *E. coli* PIR2 cells by heat shock (Hanahan, 1983). The plasmids were isolated using the Monarch Plasmid Miniprep Kit (New England Biolabs, Ipswich, MA, United States) and validated by Sanger sequencing performed by Eurofins Genomics (Ebersberg, Germany). Afterward, the plasmids were transferred to *P. putida* KT2440 via conjugation. Triparental mating using a streamlined method as outlined by Wynands et al. (2018) was performed. After mating procedures, *P. putida* strains were selected on cetrimide agar (Sigma-Aldrich, St. Louis, MO, United States). For restriction, plasmid pSW-2 was used and no 3-methylbenzoate for induction of I-SceI expression was required. Deletion mutants were verified by colony PCR using OneTaq 2x Master Mix with Standard Buffer and primers IB-282 and IB-283. At last, the recombinant strains were cured of pSW-2 plasmid by re-inoculation in LB medium without antibiotics.

To obtain biosurfactant (HAA and mono-rhamnolipid) producing strains, the mini-Tn7 delivery transposon vector developed by Zobel et al. (2015) was used. The biosurfactant synthesis module is inserted in a single genomic locus of the chromosome (*att*Tn7). The plasmid pSK02 harboring the *rhlAB* genes from *P. aeruginosa* PA01 was integrated into the genome of *P. putida* KT2440 E1.1 via transposition. Identification and selection of biosurfactant producing clones was performed as described by Bator et al. (2020), using ethanol as substrate instead of glucose.

### Analytical Methods

#### Analysis of Bacterial Growth

Bacterial growth was determined by measuring the optical density at 600 nm (OD_600_) with the Ultrospec 10 cell density meter (Biochrom, Cambridge, United Kingdom). A correlation between OD_600_ and cell dry weight (CDW) was generated. An OD_600_ of 1.0 corresponds with a CDW of 313 mg L^–1^.

#### Analysis of Metabolites and Products

Sample preparation and analysis via high performance liquid chromatography (HPLC) for the measurement of ethanol and extracellular metabolites, e.g., acetate, was performed with the same method for glucose and other organic acids as described in Bator et al. (2020). It was used a Metab-AAC 300 × 7.8 mm separation column (particle size: 10 μm, ISERA GmbH, Düren, Germany). For the measurement of HAAs and mono-rhamnolipids, sample preparation and analysis via HPLC was performed according to Bator et al. (2020) using a NUCLEODUR C18 Gravity 150 × 4.6 mm separation column (particle size: 3 μm, Macherey-Nagel GmbH & Co., KG, Düren, Germany).

### Fermentation Conditions

**FIGURE 1:**
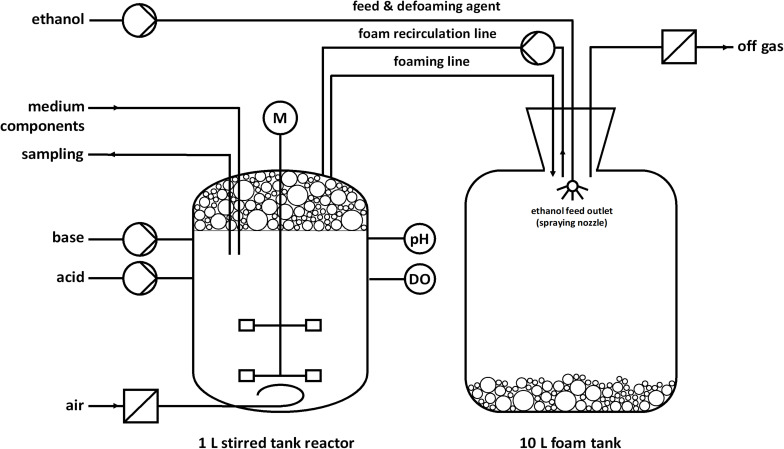
Schematic setup for a fed-batch fermentation in a bioreactor with foam recirculation and pure ethanol as feed and defoaming agent. Foam is produced during the fermentation process and collected in a foam tank. Ethanol was automatically sprayed inside the foam tank based on a DO-based feeding protocol. After the addition of ethanol, the collected foam collapsed in the tank and was continuously pumped back as a liquid.

Fed-batch fermentations for rhamnolipid production were performed as duplicate using modified M9 minimal medium with a final composition (per L) of 1.21 g Na_2_HPO_4_⋅2H_2_O, 0.43 g KH_2_PO_4_, 0.5 g NaCl, 1.9 g NH_4_Cl, 2 mM MgSO_4_, 9.74 mg FeSO_4_⋅7H_2_O, 41.2 mg CaCl_2_⋅2H_2_O, 1.5 mg MnCl_2_⋅4H_2_O, 1.87 mg ZnSO_4_⋅7H_2_O, 0.3 mg H_3_BO_3_, 0.25 mg Na_2_MoO_4_⋅2H_2_O, 0.15 mg CuCl_2_⋅2H_2_O, 13.11 mg Na_2_EDTA⋅2H_2_O (Müller et al., personal communication), and 7.6 g ethanol (0.33 Cmol) for the batch phase. The fermentations were conducted in a stirred tank reactor (BioFlo 120 glass bioreactor; Eppendorf, Hamburg, Germany) with a working volume of 1 L and a nominal volume of 1.3 L. The fermentations were controlled by a BioFlo120 unit and DASware control software (v5.5.0; Eppendorf, Hamburg, Germany). The setup is illustrated in Figure 1. The bioreactor was equipped with a pH electrode (EasyFerm plus PHI 225, Hamilton, Reno, NV, United States), dissolved oxygen (DO) electrode (InPro 6800, Mettler-Toledo, Columbus, OH, United States), sparger, pH control agent inlets, foam outlet, foam recirculation inlet, septum, Pt100 temperature sensor, hose connection for the inoculation bottle, sampling system, and cooling loop. The agitator shaft was equipped with two Rushton turbines (Ø 5.3 cm). The temperature was held constant at 30°C and the pH was adjusted to 7 via automatic addition of 30% NH_4_OH and 2 M H_2_SO_4_. NH_4_OH simultaneously served as nitrogen source. The aeration rate was 0.1 L min^–1^ (0.1 vvm), whereas evaporation was reduced by sparging the supply of sterile air through a sterile water bottle. The DO was kept above 30% by an automatic increase of the agitation speed until 1,200 rpm. The foam produced during the fermentation was directed through the foam outlet via a hose into a downstream foam collection bottle. The reactor was inoculated with cells from a preculture to a final OD_600_ of 0.5. One hour after inoculation, the peristaltic pump for foam recirculation was turned on at 220 rpm (Ø of the inner hose = 1 cm) and the stirrer speed was manually increased from 300 to 500 rpm to obtain wet foam. After the batch phase, the feeding phase was initiated. The feed was realized with an automated DO-based feeding strategy via a peristaltic pump. The feed contained 100% (v/v) ethanol (Th. Geyer GmbH & Co. KG, Renningen, Germany) and was activated at DO-levels higher than 40% and deactivated at DO-levels lower than 30%. In one feeding event, 3 mL ethanol were pumped to avoid toxic concentrations. However, the feed did not enter the reactor directly but was sprayed into the foam tank to collapse the collected foam. The collapsed foam is pumped back into the reactor by a peristaltic pump. The stirrer speed was constant at 1,200 rpm and up to 90% pure oxygen was manually mixed to the supply air. When the growth rate decreased, medium components were added to maintain growth and avoid nutrition limitation at higher cell densities. At 20 h the last supplementation was done and the medium contained in total (per L) 7.2 g Na_2_HPO_4_⋅2H_2_O, 2.58 g KH_2_PO_4_, 0.5 g NaCl, 1.9 g NH_4_Cl, 9.51 g NH_4_OH, 26 mM MgSO_4_, 48.78 mg FeSO_4_⋅7H_2_O, 0.22 g CaCl_2_⋅2H_2_O, 48.7 mg MnCl_2_⋅4H_2_O, 10.85 mg ZnSO_4_⋅7H_2_O, 3.37 mg H_3_BO_3_, 1.55 mg Na_2_MoO_4_⋅2H_2_O, 1 mg CuCl_2_⋅2H_2_O, and 65.7 mg Na_2_EDTA⋅2H_2_O.

To characterize the fed-batch fermentation results, the values of biomass, biosurfactants, and ethanol (during batch phase) were fitted using a logistic equation with four parameters as described by Wittgens et al. (2011). An independent fit was used to illustrate the development of all three fermentation parameters.

### Flux Balance Analysis

Flux balance analysis (FBA) has been carried out as described previously by Bator et al. (2020). Briefly, the genome-scale model of *P. putida* KT2440, *i*JN1463 (Nogales et al., 2020), was used and extended by the biosynthesis route for mono-rhamnolipid production. All simulations were carried out in MATLAB (version R2019b; The MathWorks, Inc., Natick, MA, United States). The uptake of ethanol was set to 10 mmol g_*CDW*_^–1^ h^–1^ and the uptake of other carbon sources (e.g., glucose) was set to zero. The maximization of production of mono-rhamnolipids or biomass was used as objective function in the extended model.

## Results

### Evolutionary Engineering Improves Growth of *P. putida* KT2440 on Ethanol

**FIGURE 2:**
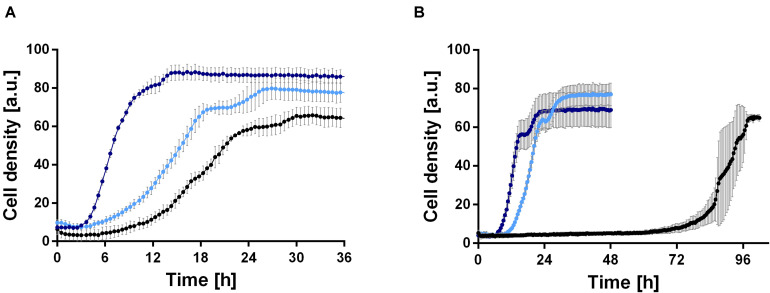
Comparison of growth of *P. putida* KT2440 (black) and two evolved strains, *P. putida* KT2440 E1 (light blue) and *P. putida* KT2440 E1.1 (dark blue), in M9 minimal medium containing ethanol. **(A)** Growth in medium containing 0.96% (v/v) ethanol and **(B)** growth in medium containing 4% (v/v) ethanol. Growth was detected using a Growth Profiler 960 in 96-well plates. Error bars indicate the standard deviation from the mean (*n* = 3).

In order to establish a rhamnolipid production process based on ethanol, the growth behavior of *P. putida* KT2440 was investigated in minimal medium with 0.96, 2, 4, 6, and 8% (v/v) ethanol as sole carbon source. 0.96% (v/v) ethanol was included, since this equals 0.33 Cmol, which is the carbon content used in all further experiments in this study. Interestingly, the wild type was able to grow natively in medium containing up to 4% (v/v) ethanol (Figure 2B). However, cell clumping was observed, indicating cell stress. The wild type exhibits a long lag phase of about 70 h at 4% (v/v) ethanol (Figure 2B), which is likely caused by the inhibitory effect of the high ethanol concentration. While even at 0.96% (v/v) cell clumping was observed, the lag phase was significantly reduced to 6 h and a growth rate of 0.21 ± 0.03 h^–1^ was reached (Figure 2A). However, the growth rate showed a sharp drop after approximately 16 h. In general, the results show that *P. putida* KT2440 is able to utilize ethanol, which was not surprising since an ethanol oxidation system and several alcohol and aldehyde dehydrogenases exist in *Pseudomonas* species (Nelson et al., 2002; Görisch, 2003).

Because the cells clumped and had a moderate growth rate, we decided to improve these traits by ALE. ALE is a technique frequently used to improve features of microbes by adaptation to various environmental conditions, such as temperatures or non-preferred carbon sources. A first ALE experiment was performed using M9 minimal medium with 2 g L^–1^ glucose and 4% (v/v) ethanol to increase the solvent tolerance for 30 days corresponding to ∼115 generations. Single colonies were obtained from the inhomogeneous culture, which were tested for growth (data not shown). The best-adapted colony (the strain with the highest growth rate and shortest lag phase after ALE) was designated *P. putida* KT2440 E1, which was used for a second ALE experiment in M9 minimal medium with 4% (v/v) ethanol for twelve days corresponding to ∼37 generations. Again, single colony isolation, and growth rate determination was used; the best adapted strain was designated *P. putida* KT2440 E1.1. After ALE, both strains showed reduced or no cell clumping in the presence of ethanol, which might correlate with ethanol tolerance. The growth of the two adapted strains was investigated in M9 minimal medium containing 0.96 or 4% (v/v) ethanol (Figures 2A,B). On 4% (v/v) ethanol, a significantly decreased lag phase of 9 and 6 h was observed for *P. putida* KT2440 E1 and *P. putida* KT2440 E1.1, respectively (Figure 2B). *P. putida* KT2440 E1 had a slightly reduced growth rate of 0.17 ± 0.02 h^–1^, while no drop of the growth rate when compared to the wild type in 0.96% (v/v) ethanol was observed. The growth rate of *P. putida* KT2440 E1.1 was increased to 0.51 ± 0.03 h^–1^.

**FIGURE 3:**
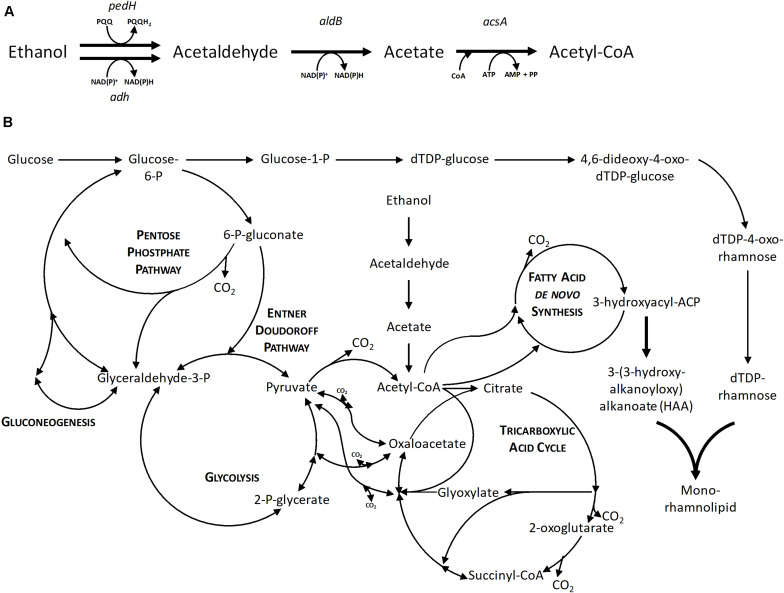
The metabolic network of *P. putida* KT2440. **(A)** Enzymatic steps of ethanol utilization. **(B)** Central carbon metabolism of *P. putida* KT2440 based on Sudarsan et al. (2014) with the native ethanol utilization pathway and the heterologous mono-rhamnolipid production pathway. Bold arrows indicate the recombinant enzymatic steps toward mono-rhamnolipid production. *pedH*, quinoprotein ethanol dehydrogenase; *adh*, alcohol dehydrogenase; *aldB*, aldehyde dehydrogenase; *acsA*, acetyl-CoA synthetase; AMP, adenosine monophosphate; ADP, adenosine diphosphate; ATP, adenosine triphosphate; NAD^+^, nicotinamide adenine dinucleotide; NADH, reduced nicotinamide adenine dinucleotide; PQQ, pyrroloquinoline quinone; PQQH_2_, reduced pyrroloquinoline quinone.

Additionally, the formation of the intermediate acetate was examined. The intermediate acetaldehyde could not be measured due to its volatile character. The enzymatic steps and introduction in the central carbon metabolism are shown in Figure 3. Samples taken until 28 h of the cultivation of *P. putida* KT2440 using 0.96% (v/v) ethanol could exclude the presence of acetate. Hence, it is hypothesized that the conversion of acetaldehyde to acetate is the rate-limiting step and instead, an acetaldehyde accumulation took place, which caused growth inhibition (drop of the growth rate after 16 h in Figure 2A) because aldehydes are highly toxic also at low levels. The evolved strains *P. putida* KT2440 E1 and E1.1 grew to higher cell densities, but they also showed higher acetate concentrations (3.1 ± 0.3 and 3.8 ± 0.1 g L^–1^), respectively. Due to ALE, the overall concentrations of the intermediates were not growth-inhibiting anymore. As a consequence, the evolved strains were able to use ethanol and acetaldehyde more efficiently indicated by the higher biomass production. However, another imbalance was revealed after ALE, which resulted in a stronger acetate accumulation through a slow conversion of acetate to acetyl-CoA. Here, a higher growth rate on ethanol and a higher ethanol tolerance were achieved. The time period of the ALE process might be extended in order to select isolates with even more improved features, i.e., a higher growth rate and lower accumulation of intermediates. However, a short time period was chosen to minimize the appearance of random background mutations, simplifying the allocation of mutations to the observed phenotypes.

### Rerouting of Metabolism for Carbon-Efficient Ethanol Utilization Caused by Adaptation

**FIGURE 4:**
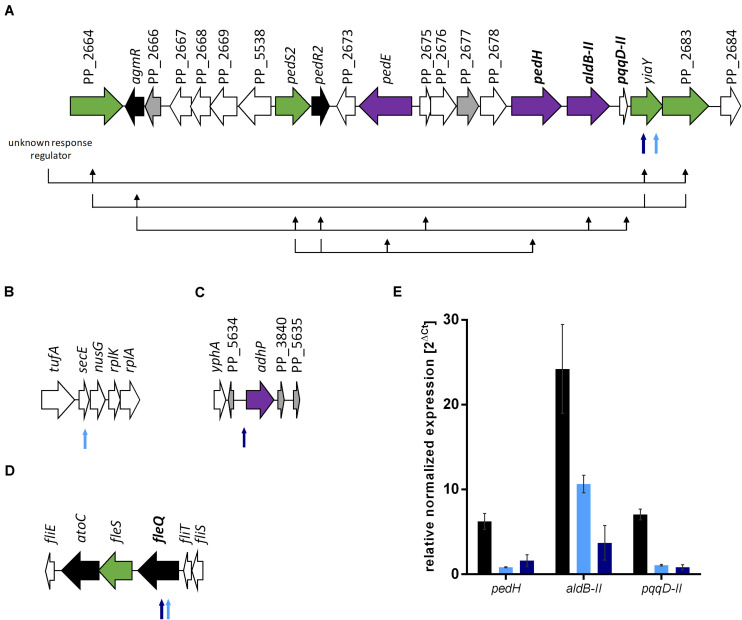
Genomic consequences of ALE on gene expression. The gene organization is based on the genome sequence AE015451.2 (Belda et al., 2016). **(A)** Gene organization from PP_2664 to PP_2684 with a schematic hypothetical regulatory scheme below the genes based on regulation patterns in *P. aeruginosa* (Hempel et al., 2013). The black arrows display which genes have a positive regulatory influence on other genes. Further unidentified two-component systems genes are probably involved but not considered in this figure. The genes in bold font were investigated for gene expression levels. **(B)** Gene organization from *tufA* to *rplA* (PP_0440 to PP_0444). **(C)** Gene organization from *yphA* (PP_3837) to PP_5635. **(D)** Gene organization from *fliE* to *fliS* (PP_4370 to PP_4375). **(E)** Relative normalized expression of genes involved in ethanol metabolism of *P. putida* KT2440 (black bars), *P. putida* KT2440 E1 (light blue), and *P. putida* KT2440 E1.1 (dark blue) growing on 0.96% (v/v) ethanol. The 2^Δ*Ct*^ values were normalized to *rpoB* and error bars represent the maximum and minimum values of two independent measurements (*n* = 2). Black arrows indicate genes coding for regulators, green arrows indicate genes coding for sensor kinases, purple arrows indicate genes involved in ethanol conversion, gray arrows indicate hypothetical proteins, and white arrows indicate genes with other functions. Smaller arrows below the gene organization show the identified mutations after ALE. The light blue arrows indicate the presence of the mutation in *P. putida* KT2440 E1 and the dark blue arrows indicate the presence of the mutation in *P. putida* KT2440 E1.1.

To investigate the basis of the phenotypic changes after ALE, genome re-sequencing of both evolved strains and *P. putida* KT2440 as reference was performed. Furthermore, the metabolic network structure was examined by FBA. We could identify 60 mutations, including 36 SNPs and 24 insertion-deletion polymorphisms (InDel) for the reference strain *P. putida* KT2440 from our stain collection compared to the genome sequence of *P. putida* KT2440 (AE015451.2; Belda et al., 2016). This phenomenon was observed before in other *P. putida* KT2440 genome re-sequencing studies (Belda et al., 2016; Li et al., 2019). Similar mutation patterns of two reference strains from different laboratories were observed, even though the reference strains share no common history (Li et al., 2019). Most of the mutations are located in non-coding regions, silent mutations, or errors due to low coverage and read quality. Seven additional SNPs and one InDel were identified for the evolved E1. *P. putida* KT2440 E1.1 carried three SNPs and six InDels in addition to E1. Four genomic regions, which probably have an influence on ethanol tolerance and utilization were identified (Figure 4). More specifically, all SNPs and InDels of strains E1 and E1.1 were considered except silent mutations, mutations in hypothetical proteins, mutations with low coverage and read quality, and mutations in non-coding regions (except one mutation in a probable promoter region). Three mutations were found in strain E1 and three additional mutations in strain E1.1 (Supplementary Table 2). Two physiological effects can be distinguished: enhanced ethanol tolerance and more efficient ethanol utilization (higher ethanol uptake rate).

**FIGURE 5:**
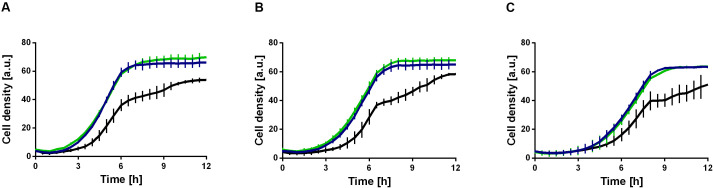
Ethanol tolerance of *P. putida* KT2440 and derivatives in M9 minimal medium containing different ethanol concentrations. *P. putida* KT2440 (black), *P. putida* KT2440 E1.1 (blue), and *P. putida* KT2440 Δ*fleQ* (green). **(A)** Minimal medium containing 10 g L^–1^ glucose and 1% (v/v) ethanol. **(B)** Minimal medium containing 10 g L^–1^ glucose and 2% (v/v) ethanol. **(C)** Minimal medium containing 10 g L^–1^ glucose and 3% (v/v) ethanol. Growth was detected using a Growth Profiler 960 in 96-well plates. Error bars indicate the standard deviation from the mean (*n* = 3).

The mutations, which might effectuate a higher ethanol tolerance, are present in both strains and were thus introduced in the first evolution round (Figures 4B,D). These include a missense mutation in *secE*, which encodes a protein translocase subunit, and a nonsense mutation in *fleQ*, which encodes a transcriptional regulator for flagellar and biofilm formation. The presence of organic solvents leads to altered lipid compositions and leakage of the plasma membrane in microbes as reviewed by Heipieper et al. (1994). The mutation in *secE* was perhaps introduced to counterbalance this effect due to an altered translocation of proteins across and insertion into the membrane. Furthermore, the nonsense mutation in *fleQ* likely causes the gene function to be negatively affected. In *P. putida* KT2440 E1.1, a frameshift was additionally introduced in *fleQ* to abolish the gene function. In recent studies, it was reported that *fleQ*-deficient *P. putida* strains showed a defect in swarming and reduced biofilm formation, which is caused by a lower expression of flagellar- and biofilm-related genes (Yousef-Coronado et al., 2008; López-Sánchez et al., 2016; Navarrete et al., 2019). Further, a relation between the disruption of flagellar-related genes and solvent tolerance in *P. putida* was described (Domínguez-Cuevas et al., 2006; Molina-Santiago et al., 2017). To verify this hypothesis, the gene *fleQ* was deleted. This deletion mutant, the reference strain, and the evolved strain E1.1 were then cultivated in minimal medium containing glucose and increasing ethanol concentrations. *P. putida* KT2440 Δ*fleQ* and *P. putida* KT2440 E1.1 showed an identical growth behavior when concentrations from 1 to 3% (v/v) of ethanol were present (Figure 5). In contrast, the reference strain showed even in the presence of low ethanol concentrations a prolonged lag phase and decreased growth compared to the deletion mutant and the evolved strain (Figure 5). With the *fleQ* deletion we could show that i) the mutations in *fleQ* are likely to contribute to an increased ethanol tolerance and ii) the mutations indeed in all likelihood lead to a deactivation of regulation of flagellar and biofilm formation.

Further mutations, which are likely assigned to ethanol utilization, were identified. Two mutations were found in a gene, of which the gene product is likely involved in the regulation of the ethanol oxidation system, which is encoded by genes localized in an operon (Figure 4A) (Görisch, 2003; Belda et al., 2016). A missense mutation was introduced in *yiaY* (PP_2682) and in the longer evolved strain an additional nonsense mutation was introduced upstream in the same gene (Supplementary Table 2). The gene *yiaY* is proposed to encode a Fe-containing alcohol dehydrogenase. A genome analysis using BLAST (Altschul et al., 1990) showed that *yiaY* (PP_2682) and PP_2683 (a two-component system sensor histidine kinase/response regulator) of *P. putida* KT2440 have sequence similarities of 84% to *ercA* (PA1991) and 72% to *ercS* (PA1992) of *P. aeruginosa* PAO1, respectively. The gene *ercA* forms an operon with *ercS* and the gene product is presumed to generate a signal that activates the sensor kinase ErcS. This has an impact on the regulator AgmR, which controls the transcription of genes encoding components for ethanol oxidation (Figure 4A). Consequently, the gene product of *ercA* is part of a complex regulatory system and controls the expression of the quinoprotein ethanol oxidation genes in *P. aeruginosa* (Gliese et al., 2004; Hempel et al., 2013). Since the synteny of the ethanol oxidation system in *P. putida* and *P. aeruginosa* share similarities, we assumed that *yiaY* has a similar function in *P. putida* KT2440. To confirm this assumption, gene expression levels of the presumably regulated genes *pedH*, *aldB-II*, and *pqqD-II*, which are involved in ethanol oxidation (Figure 4A), in *P. putida* KT2440 and the two evolved strains E1 and E1.1 were determined via qRT-PCR. Interestingly, the transcript level of all three genes was highest for the reference strain (Figure 4E). *P. putida* KT2440 E1 had a lower transcript level of the regulated ethanol oxidation genes compared to the reference strain and the transcript levels of these genes of strain E1.1 were even lower. These findings indicate that *yiaY* is involved in the regulation of expression of ethanol oxidation genes since no other mutations were found in the surrounding kinases or regulators. Further, a low transcription of the genes involved in ethanol oxidation (*pedH*, *aldB-II*, and *pqqD-II*) could be observed in *P. putida* KT2440 E1.1, although a stop codon was introduced in *yiaY*. The stop codon was present in the second half of the gene and thus, there might be a residual activity. As mentioned before, the ethanol oxidation system is regulated by a complex regulatory system and other factors also might still regulate the transcription of the genes positively. However, it was observed that the transcript levels of the ethanol oxidation genes in the operon were more decreased the longer the strain was evolved by ALE. This fits the hypothesis, which was formulated in the beginning based on the growth of the strains. It was argued that the overall concentration of the intermediates was not growth-inhibiting anymore and that an adaptation to the intermediates happened. These changes were the basis for more efficient use of the substrate. The gradual reduction of transcript identified by qRT-PCR entailed a slower conversion of ethanol and thus, lower concentrations of the toxic intermediate acetaldehyde might be present. Accumulation of acetaldehyde was suggested as a reason for the sharp drop of the growth rate for the wild type, which was not present in both evolved strains. In strain E1, acetaldehyde accumulation might be reduced below a toxic level for the cells enabling the further conversion of acetaldehyde into acetate. However, acetate accumulation was observed, indicating a second bottleneck (slow conversion to acetyl-CoA). This could have led to the further decrease of transcript levels, which is probably caused by the missense mutation in *yiaY* in strain E1.1. The mutation has a reductive effect on the expression of the quinoprotein ethanol oxidation gene, *pedH*, and *aldB-II* and possibly forces the strain to convert ethanol slower.

In *P. putida* KT2440 E1.1, the transcript levels of the genes for the ethanol oxidation enzymes, located in the operon (Figure 4E), were further decreased. However, other dehydrogenases may undertake the ethanol oxidation. One mutation in a gene, which is probably involved in ethanol degradation, occurred, causing that the encoded enzyme may undertake the conversion of ethanol to acetaldehyde. A SNP in the intergenic region before *adhP* (PP_3839), encoding an alcohol dehydrogenase, which is proposed to be located in the cytoplasm, was found (Figure 4C). The mutation upstream of *adhP* might alter the promoter activity upstream of *adhP*. Using the Softberry BPROM tool (Solovyev and Salamov, 2011), different promoters were predicted for the reference strain and *P. putida* KT2440 E1.1, i.e., the −10 region of the probable promoter upstream of *adhP* is affected by the mutation (Supplementary Figure 1). Thus, the alteration caused by the mutation might have contributed to a significantly improved growth rate (Figure 2).

Although the mutations and resulting phenotypes, including the discovery of their underlying basis, were analyzed, the question why the quinoprotein dependent ethanol oxidation was downregulated and other dehydrogenases overtook their functions, are intriguing. To investigate this, FBA was used to predict fluxes and yields regarding biomass production using the genome-scale model *P. putida* KT2440 *i*JN1463 (Nogales et al., 2020). Two different scenarios, the PQQ-dependent and NAD^+^-dependent ethanol conversion, were tested. Maximal product yields of 37 mmol mol^–1^ and 41 mmol mol^–1^ were computed for the PQQ-dependent ethanol conversion and the NAD^+^-dependent ethanol conversion, respectively. To elucidate where the differences derive from, different fluxes were analyzed. In the reference strain *P. putida* KT2440 (PQQ-dependent), the electron pair is used in the respiratory chain for ATP synthesis and not for biomass production, thus a higher flux through the TCA cycle is needed to regenerate more NADH theoretically. With FBA, a higher respiration (14.7 mmol g_*CDW*_^–1^ h^–1^ O_2_) and a higher flux through the TCA cycle, represented by the conversion of citrate to *cis*-aconitate (4.87 mmol g_*CDW*_^–1^ h^–1^), compared to the NAD^+^-dependent ethanol conversion was computed. This leads to a higher CO_2_ production (5.33 mmol g_*CDW*_^–1^ h^–1^) using the PQQ-dependent ethanol conversion instead of 3.52 mmol g_*CDW*_^–1^ h^–1^ CO_2_ using the NAD^+^-dependent ethanol conversion. Thus, the changes obtained by ALE contributed to a more carbon-efficient route of ethanol degradation and led to an adaptation to the intermediates, which can explain the more efficient use of the substrate indicated by the higher biomass concentrations of the mutant strains E1 and E1.1.

### Biosurfactant Production Is Increased Due to More Efficient Substrate Utilization

**FIGURE 6:**
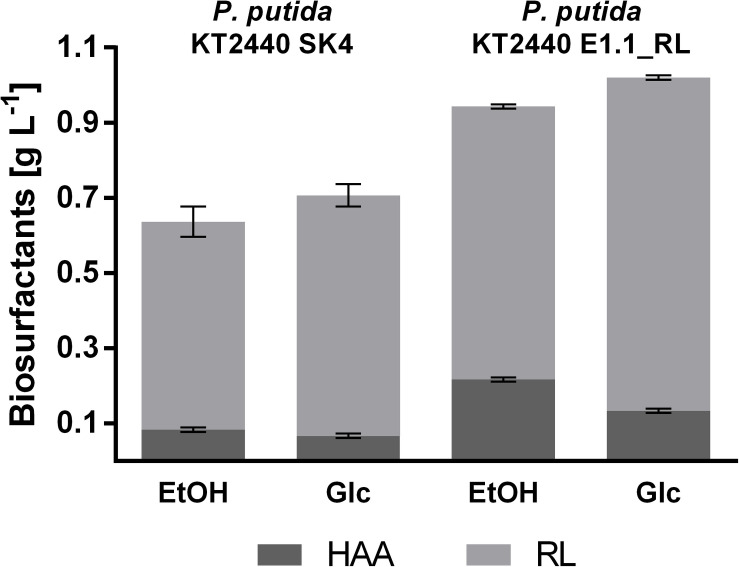
Proportion of different biosurfactants produced by *P. putida* KT2440 SK4 and *P. putida* KT2440 E1.1_RL in 24-deep well plates using M9 minimal medium. The minimal medium contained 0.96% (v/v) ethanol (EtOH) or 10 g L^–1^ glucose (Glc) (both 0.33 Cmol L^–1^). Shown are the product titers of HAAs (dark gray) and mono-rhamnolipids (RL; light gray). Error bars indicate the standard deviation from the mean (*n* = 3).

The production of mono-rhamnolipids and HAAs (from now on referred to as biosurfactants) was consequently investigated with an efficient ethanol metabolizer (*P. putida* KT2440 E1.1) and compared to the wild type in 24-deep well plates (Figure 6). For the production, the biosurfactant synthesis module was integrated as a single copy into the genome of *P. putida* KT2440 E1.1. The biosurfactant producer was designated *P. putida* KT2440 E1.1_RL. For comparison, the wild type with the integrated biosurfactant synthesis module, *P. putida* KT2440 SK4 (Tiso et al., in review), was used. The production was performed using 0.96% (v/v) ethanol or 10 g L^–1^ glucose (both 0.33 Cmol L^–1^) as the sole carbon source to compare the production capacities on ethanol and the conventionally used glucose. The cultivation was performed for 48 h to ensure complete consumption of the substrates for both strains.

The product to substrate yield for *P. putida* KT2440 SK4 was in a similar range for glucose and ethanol, while the product to biomass yield was significantly higher (1.6-fold) when ethanol was used (Table 2). Further, the distribution of the different congeners was similar and the proportion of HAAs was in a similar range for both substrates (13 and 9%), indicating that rhamnose provision is not significantly reduced when ethanol is used as a carbon source (Figure 6). The evolved strain E1.1 showed higher final biosurfactant titers, which is possibly caused by the higher biomass concentration. This also suggests that the evolved strain E1.1 uses the substrates more efficiently. Whereas the cause for the higher production on glucose remains unknown, in the case of ethanol, the rerouting of the metabolism is possibly the cause. As described before, the evolved strain downregulated the quinoprotein-dependent ethanol oxidation and instead likely used NAD^+^-dependent ethanol oxidation. With FBA not only for biomass production but also for rhamnolipid production, a higher yield using NAD^+^ as cofactor was predicted. The predicted maximal product yield using PQQ and NAD^+^ as a cofactor is 0.63 and 0.71 mmol mmol^–1^, respectively. Regarding the further evolved strain, E1.1 reached a similar product to substrate yield on ethanol compared to glucose but also reached a significantly higher product to biomass yield (Table 2). Further, the distribution of the different congeners was similar on both substrates, but a lower proportion of HAAs was produced on glucose (13%) compared to ethanol (23%) (Figure 6). This indicates a lack of activated rhamnose due to the conversion of ethanol to acetyl-CoA, which is then introduced in the tricarboxylic acid (TCA) cycle or the fatty acid *de novo* synthesis (Figure 3B). To obtain dTDP-L-rhamnose, the strain has to undertake gluconeogenesis to synthesize glucose-6-phosphate, which is energy-intensive. Since this phenomenon was only observed for *P. putida* KT2440 E1.1_RL, a reduced relative metabolic flux in the direction of hexoses after ALE is assumed yielding in a higher share of HAAs.

**TABLE 2:** Cultivation characteristics of the recombinant biosurfactant producers (CDW; cell dry weight, BS: biosurfactant (HAAs and mono-rhamnolipids), S: substrate).

Organism	Substrate	CDW (g_*CDW*_ L^–^^1^)	Maximal titer (g_*BS*_ L^–^^1^)	Product to substrate yield (Cmol_*BS*_ Cmol_*S*_^–^^1^)	Product to biomass yield (g_*BS*_ g_*CDW*_^–^^1^)
*P. putida* KT2440 SK4	Ethanol (7.6 g L^–1^)	1.11 ± 0.08	0.64 ± 0.03	0.10 ± 0.00	0.59 ± 0.07
*P. putida* KT2440 SK4	Glucose (10 g L^–1^)	1.94 ± 0.03	0.71 ± 0.03	0.11 ± 0.00	0.36 ± 0.01
*P. putida* KT2440 E1.1_RL	Ethanol (7.6 g L^–1^)	1.63 ± 0.05	0.94 ± 0.01	0.15 ± 0.00	0.58 ± 0.01
*P. putida* KT2440 E1.1_RL	Glucose (10 g L^–1^)	2.42 ± 0.05	1.02 ± 0.01	0.16 ± 0.00	0.42 ± 0.01

The product to biomass yield for each substrate is comparable for *P. putida* KT2440 SK4 and *P. putida* KT2440 E1.1_RL, with a higher product to biomass yield for ethanol. This points out that using ethanol leads to a higher production capacity per cell and that the stoichiometry of the ethanol degradation pathway is superior for biosurfactant production compared to glucose. Namely, ethanol is converted to acetyl-CoA directly being introduced in the fatty acid *de novo* synthesis without carbon loss, which is the precursor pathway for rhamnolipids (Figure 3B). Further, ethanol has a high degree of reduction, which means that more electrons are available and hence more energy is conserved in the substrate compared to glucose. This contributes to achieving a higher biomass or product yield. The theoretical product to substrate yield on ethanol is 0.93 Cmol Cmol^–1^ for zero growth, of which 16% were reached with *P. putida* KT2440 E1.1_RL. Further, the theoretical product to substrate yield on ethanol is 29% higher than on glucose.

### The Use of Ethanol Enables the Development of an Efficient Biosurfactant Production Process

In order to increase the biosurfactant titer, a fed-batch fermentation procedure was developed. Biosurfactants tend to foam due to their amphiphilic structure. Thus, a fermentation process is challenging to control and often realized with additional equipment, chemicals, or process steps, which increases the costs and the effort in downstream processing. To keep the setup as simple as possible, an integrated foam recirculation with automated ethanol addition was developed.

The fermentations were conducted as biological duplicates with *P. putida* KT2440 E1.1_RL using ethanol as sole carbon source. In this setup, ethanol serves two functions. First, it is used as a carbon source for the production of biomass and biosurfactants. Second, it is used as a defoaming agent because the foam is destabilized when getting in contact with the ethanol. The collapsed foam with fresh carbon source was pumped back into the bioreactor, preventing thus loss of biomass and product while at the same time a fed-batch process is established.

**FIGURE 7:**
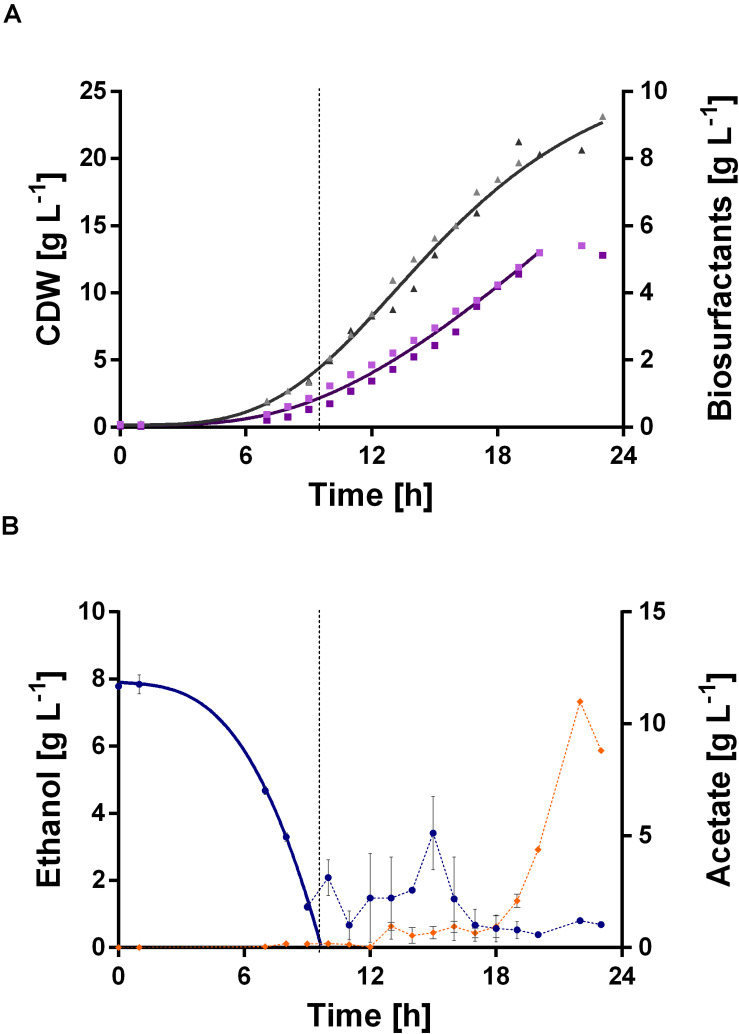
DO-triggered fed-batch fermentation for biosurfactant production with *P. putida* KT2440 E1.1_RL using ethanol as sole carbon source. The fermentation was performed as a duplicate. **(A)** CDW (▲, gray) and biosurfactant concentration (■, purple) and their respective fitted courses. The values are given as single representative values of two bioreactor cultivations. **(B)** Ethanol concentration (•, blue) and acetate concentration (◆, orange) and the respective fitted course for ethanol in the batch phase. The dashed lines indicate the switch from batch to the fed-batch phase. The values are given as mean values of two bioreactor cultivations and error bars represent the maximum and minimum values of two independent experiments.

**FIGURE 8:**
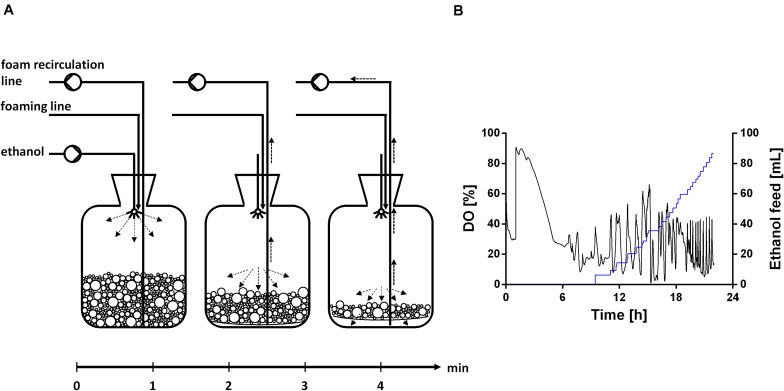
Ethanol feeding procedure. **(A)** Schematic overview of the ethanol feed in the foam collection bottle based on a DO-based feeding protocol. **(B)** DO signal (black), which is controlled by a DO-based feeding protocol, and ethanol feed volume (blue), exemplarily shown for one reactor.

The fermentation started with a batch phase [0.96% (v/v) ethanol], which ended after ethanol was consumed (9.5 h). A biosurfactant titer of about 0.7 g L^–1^ was reached after 9 h (Figure 7A). Further, a biomass concentration of around 3.5 g L^–1^ was reached (Figure 7A). The biosurfactant titer is 27% lower and the biomass concentration is 2-fold higher compared to the experiment in 24-deep well plates (Table 2). After the batch phase, the foam collection bottle was full of foam. To initiate the feed phase, 6 mL ethanol were added manually to collapse the foam. Subsequently, 3 mL ethanol were introduced automatically via the spraying nozzle by using a DO-based feeding script. The feed pulse was triggered when the DO signal increased to 40%. To account for the built-in inertia of the control loop, which is schematically demonstrated in Figure 8A, a break of 5 min was introduced after the feed trigger. During this pause, the DO signal dropped below 30%, causing the script to cease feeding. Once the DO signal reached again 40%, a new feed cycle was initialized, which can be seen in the oscillating DO signal (Figure 8B). In total, around 90 mL ethanol were fed. At the end of the fermentation a biosurfactant titer of about 5.3 g L^–1^ and biomass concentrations of about 22 g L^–1^ were reached. A growth rate of 0.14 ± 0.00 h^–1^ was reached in the feed phase, which marks a reduction by 60% compared to the batch phase. This was likely caused by carbon limitation due to the linear feeding profile (Figure 8B). With this setup, a product to substrate yield of 0.065 ± 0.006 g_*BS*_ g_*EtOH*_^–1^ was reached. The yield is 41% reduced compared to the production experiment in 24-deep well plates, which can be explained by the high biomass production, which benefits from the high oxygen and nitrogen supply. Further, a space time yield of 0.23 ± 0.01 g_*BS*_ L^–1^ h^–1^ was achieved over the whole fermentation.

Again, acetate accumulation occurred after 18 h of fermentation. At the end of the fermentation an acetate concentration of about 10 g L^–1^ was observed. This entails two issues: On the one hand, the carbon source was thus not completely available for biosurfactant production, which adulterates the yield. Since the accumulated acetate can theoretically be converted to biosurfactants, the actual yield is 0.072 ± 0.007 g_*BS*_ g_*EtOH*_^–1^. On the other hand, high acetate concentrations might cause growth inhibition. Contrary to the flask experiments of *P. putida* KT2440 E1.1, where approximately 3.8 g L^–1^ acetate accumulated, here no acetate was present after the batch phase. This might be caused by the introduction of additional metabolic demand for β-hydroxy fatty acids and thus acetyl-CoA (via biosurfactant production) and hence the increased conversion of acetate to acetyl-CoA.

This fermentation setup demonstrates the potential of using ethanol concomitantly as an efficient defoamer and carbon source. By automated ethanol addition, the supply of carbon is assured, resulting in an increase of biomass and biosurfactants throughout the whole fermentation. Additionally, no further equipment, such as mechanical foam breakers or antifoam, were necessary to control the foam.

Here, we show that the combination of strain and process design in an early stage resulted in the development of a novel fermentation setup. Strain engineering facilitated the use of ethanol, which subsequently could be used as foam-controlling agent and carbon source.

## Discussion

Taken together, the results show that a fed-batch rhamnolipid fermentation process can be developed without using conventional antifoam or mechanical foam breakers. To achieve this, we evolved *P. putida* KT2440 to an efficient ethanol metabolizer. The evolved strain was then characterized regarding growth and heterologous rhamnolipid production and was used to develop a unique rhamnolipid production process.

Considering the metabolic network configuration, ethanol is an ideal substrate for rhamnolipid production because it is converted to acetyl-CoA without carbon loss and feeds directly in the fatty acid *de novo* synthesis or the TCA cycle. Thereby, the formation of by-products derived from pyruvate can be circumvented and the yield may be increased. In addition, ethanol degradation delivers energy in form of NADH. This characteristic was already exploited for mevalonate production in *P. putida* KT2440 and rhamnolipid production in *P. aeruginosa* (Matsufuji et al., 1997; Santos et al., 2002; Yang et al., 2019). Moreover, bioethanol can be produced from alternative non-food substrates, e.g., lignocellulosic biomass (Wendisch et al., 2016; Duwe et al., 2019). Thus, the use of ethanol as a carbon source contributes to establishing a circular bioeconomy while also creating an advantage in terms of developing a long-lasting fermentation process. Ethanol has a defoaming property, which was described in a rhamnolipid production process using *P. aeruginosa* (Sha et al., 2012). While the ethanol has no defoaming or antifoaming effect in the fermentation broth, foam in a foam collection bottle collapses if ethanol is sprayed on it. Therefore, a setup with foam collection and recirculation was chosen (Figure 1). Sha et al. (2012) used a similar setup but used colza oil as substrate and manually added ethanol to collapse the collected foam. While the addition of ethanol before 48 h decreased the rhamnolipid and biomass production in their case, in our study, ethanol concomitantly served as carbon source and defoamer. The presence of ethanol to maintain growth and rhamnolipid production and collapsing of foam was ensured by a DO-based feeding script and hence occurred automatically. This setup theoretically allows the continuation of the fermentation beyond the batch phase for several days. Further, this study demonstrates the potential of combining strain and process development already in the early stages of process development. This integrated strain and process engineering approach was strongly emphasized by Kuhn et al. (2010), while consecutive optimization of strain and fermentation is still the norm. Engineering a biosurfactant producer that is able to tolerate high ethanol concentrations and utilize ethanol as carbon source unlocked the usage of the carbon source also as defoaming agent. The general strategy that the carbon source acts as antifoam is prominent in conventional rhamnolipid production using plant oils as carbon source. However, ethanol features the striking advantage of being water-soluble, which significantly reduces subsequent downstream processing efforts.

Compared to previous reports about recombinant rhamnolipid production with *P. putida* KT2440, the here obtained results using *P. putida* KT2440 as host for heterologous rhamnolipid production are comparable or even superior. In this fed-batch fermentation, a product to substrate yield of 0.072 g_*BS*_ g_*EtOH*_^–1^ and a space time yield of 0.23 g_*BS*_ L^–1^ h^–1^ was reached. In a study with an integrated rhamnolipid adsorption system using glucose as carbon source, a yield of 0.05 g_*RL*_ g^–1^ and a space time yield of 0.073 g_*RL*_ L^–1^ h^–1^ were reached (Anic et al., 2018). Beuker et al. (2016a) reported a titer of 14.7 g L^–1^, which corresponds to a yield of 0.09 g_*RL*_ g^–1^ and a space time yield of 0.2 g_*RL*_ L h^–1^ in a fed-batch fermentation using glucose and antifoam to control the foaming. With the native rhamnolipid producer *P. aeruginosa* a titer of 36.7 g_*RL*_ L^–1^ was achieved with a yield of 0.15 g_*RL*_ g^–1^ in a fermentation with foam separation using sunflower oil (Müller et al., 2011). While in general, hydrophobic substrates are known to generate the best product yields (Nitschke et al., 2011), also other carbon sources were already investigated. A fed-batch cultivation of *P. aeruginosa* using a medium supplemented with yeast extract and ethanol resulted in 32 g_*RL*_ L^–1^ rhamnolipids, which corresponds to a yield of 0.58 g_*RL*_ g^–1^ (Matsufuji et al., 1997). Hence, further improvements are needed to reach yields and titers comparable to those of *P. aeruginosa* with the here described setup.

Improvements envisaged are: First, a higher proportion of HAAs was produced by the evolved *P. putida* KT2440 E1.1_RL strain on ethanol (Figure 6), which indicates a bottleneck in the availability of the precursor dTDP-L-rhamnose. This might be diminished by the addition of hexoses as glucose, since glucose is directly used in the rhamnose pathway to yield dTDP-L-rhamnose (Rahim et al., 2000). Metabolic pathways for utilization of various substrates have natural stoichiometries leading to biosynthetic imbalance and suboptimal product yields (Park et al., 2019). Thus, using a mixture of substrates with introduction in several pathways may meet the requirements for optimal biosynthesis. However, some substrates are preferred and thus preventing the co-metabolization of less preferred substrates. This challenge was addressed by Park et al. with a synergistic substrate cofeeding approach, where a controlled limited feeding of glucose as dopant enabled a co-metabolization of acetate and glucose and enhanced product synthesis. Especially the use of least preferred carbon sources, such as CO_2_ or acetate, can be facilitated with such an approach contributing to a sustainable bioeconomy (Park et al., 2019). Second, acetate accumulation in the fed-batch fermentation after 18 h was observed (Figure 7B). A concentration of over 10 g L^–1^ acetate likely caused growth inhibition. Recently, Arnold et al. (2019) tested 10 g L^–1^ acetate as the sole carbon source for rhamnolipid production with a recombinant *P. putida* KT2440 and also identified a growth inhibition. Further, an acetate accumulation during growth on ethanol was reported for *P. putida* KT2440 (Yang et al., 2019). The authors hypothesized that the oxidation of ethanol to acetate is rapid and that the rate of acetate conversion to acetyl-CoA is limited and thus acetate accumulates. Yang et al. produced mevalonate using ethanol and observed acetate accumulation for a strain carrying the empty plasmid, while no acetate was present when the strain produced mevalonate. This corresponds with our results from batch cultivations, promoting our hypothesis that there is an additional metabolic demand for acetyl-CoA when thereof derived products are synthesized.

In summary, we report rhamnolipid production with a recombinant *P. putida* KT2440 derivative using ethanol as an alternative carbon source and defoamer, increasing substantially the possibilities in the fermentation set-up.

## Data Availability Statement

The raw data supporting the conclusions of this article will be made available by the authors, without undue reservation, to any qualified researcher.

## Author Contributions

IB performed all the engineering and characterization experiments, prepared the figures, and wrote the manuscript. TK performed the fermentations with the help of IB. TT and LB conceived the study, advised on all the experiments, discussed the data, and edited the manuscript. All authors contributed to the article and approved the submitted version.

## Conflict of Interest

LB and TT declare that they are inventors of three related patents. (1) LB, F. Rosenau, S. Wilhelm, A. Wittgens, TT, “Means and methods for rhamnolipid production” HHU Düsseldorf University, TU Dortmund University, 2013 (WO 2013/041670 A1), (2) LB, B. Küpper, E. M. del Amor Villa, R. Wichmann, C. Nowacki, “Foam adsorption” TU Dortmund University, 2013 (WO 2013/087674 A1), and (3) LB, TT, A. Germer, “Extracellular production of designer hydroxyalkanoyloxy alkanoic acids with recombinant bacteria” RWTH Aachen University, 2015 (WO2017006252A1). The remaining authors declare that the research was conducted in the absence of any commercial or financial relationships that could be construed as a potential conflict of interest.

## Footnotes

1 http://primer3.ut.ee
