# Corrigendum: Killing Two Birds With One Stone – Strain Engineering Facilitates the Development of a Unique Rhamnolipid Production Process

**DOI:** 10.3389/fbioe.2020.596414

**Published:** 2020-10-07

**Authors:** Isabel Bator, Tobias Karmainski, Till Tiso, Lars M. Blank

**Affiliations:** ^1^iAMB – Institute of Applied Microbiology, ABBt – Aachen Biology and Biotechnology, RWTH Aachen University, Aachen, Germany; ^2^Bioeconomy Science Center (BioSC), Forschungszentrum Jülich, Jülich, Germany

**Keywords:** *Pseudomonas*, metabolic engineering, synthetic biology, adaptive laboratory evolution, ethanol, rhamnolipid, fermentation, biosurfactants

In the original article, there was a mistake in Figure 5 as published. A wrong graph was shown in part B. The corrected Figure 5 appears below.

**Figure 5:**
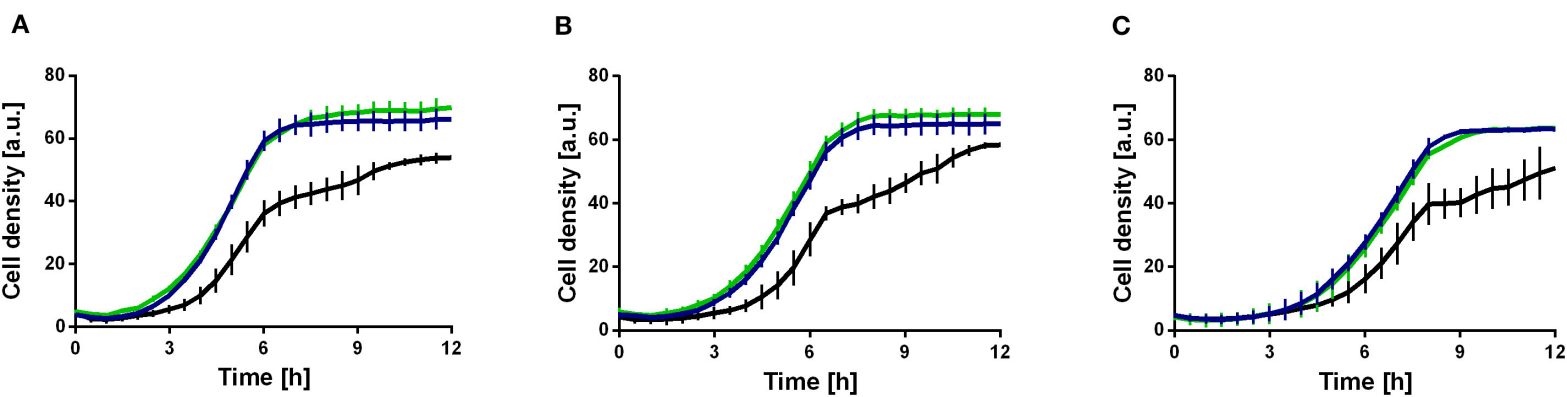
Ethanol tolerance of *P. putida* KT2440 and derivatives in M9 minimal medium containing different ethanol concentrations. *P. putida* KT2440 (black), *P. putida* KT2440 E1.1 (blue), and *P. putida* KT2440 Δ*fleQ* (green). **(A)** Minimal medium containing 10 g L^−1^ glucose and 1% (v/v) ethanol. **(B)** Minimal medium containing 10 g L^−1^ glucose and 2% (v/v) ethanol. **(C)** Minimal medium containing 10 g L^−1^ glucose and 3% (v/v) ethanol. Growth was detected using a Growth Profiler 960 in 96-well plates. Error bars indicate the standard deviation from the mean (*n* = 3).

The authors apologize for this error and state that this does not change the scientific conclusions of the article in any way. The original article has been updated.

