# Supplementary material for: Killing Two Birds With One Stone – Strain Engineering Facilitates the Development of a Unique Rhamnolipid Production Process
Source: Front Bioeng Biotechnol. 2020 Aug 7;8:899. doi: 10.3389/fbioe.2020.00899 (PMC7427536; doi:10.3389/fbioe.2020.00899)
Supplement: Supplementary file 1 [file Data_Sheet_1.docx]

Supplementary Material

Killing two birds with one stone – strain engineering facilitates the development of a unique rhamnolipid production process

Isabel Bator^1,2^, Tobias Karmainski^1,2^, Till Tiso^1,2^*, Lars M. Blank^1,2^*

^1^iAMB - Institute of Applied Microbiology, ABBt – Aachen Biology and Biotechnology, RWTH Aachen University, Aachen, Germany

^2^Bioeconomy Science Center (BioSC), Forschungszentrum Jülich, Jülich, Germany

*** Correspondence:** Till Tiso till.tiso@rwth-aachen.de, Lars M. Blank lars.blank@rwth-aachen.de

# Supplementary Figures and Tables

**Supplementary Table 1:** List of oligonucleotides used in this work.

| Name | Direction | Used for | Sequence |
| --- | --- | --- | --- |
| IB-5  IB-6  IB-278  IB-279  IB-280  IB-281  IB-282  IB-283  IB-311  IB-312  IB-313  IB-314  IB-315  IB-316  rpoB f  rpoB r | fwd  rev  fwd  rev  fwd  rev  fwd  rev  fwd  rev  fwd  rev  fwd  rev  fwd  rev | pEMG-*fleQ*  pEMG-*fleQ*  *P. putida* gDNA  *P. putida* gDNA  *P. putida* gDNA  *P. putida* gDNA  *P. putida* gDNA  *P. putida* gDNA  *P. putida* cDNA  *P. putida* cDNA  *P. putida* cDNA  *P. putida* cDNA  *P. putida* cDNA  *P. putida* cDNA  *P. putida* cDNA  *P. putida* cDNA | CAAGGCGATTAAGTTGGG  TCCGGCTCGTATGTTGTG  taacagggtaatctgaattcCACCCCGCCAGGCAGCAA  ttgctattgcTGTTTGGGCTGGGTGTTCGC  agcccaaacaGCAATAGCAACTTCCCTAGTCATATC  ttgcatgcctgcaggtcgacGCCTCCGCCGAGCAATAATAC  CAATTGGCCGATCAGAGG  GAAGTCGCGCAGTTGATG  GGACATCTGGAACGAAGGCA  AGCACGCCGATGTAGTCTTC  GTTCATGGCCGAGGTGATGA  TGTCGTACTGTTGCTCGGAC  GGTCCTCGAGCTGCTGGA  CAGTACGTCCTCTTCGAGCC  ATACGGCGCGGCATACA  CGTTCACGTCGTCCGACTT |

Supplementary Table 2: Identified mutations after genome re-sequencing of *P. putida* KT2440 E1 and *P. putida* KT2440 E1.1

| **Strain** | **Region** | **Position** | **Mutation** | **Outcome** |
| --- | --- | --- | --- | --- |
| *P. putida* KT2440 E1  *P. putida* KT2440 E1.1 | PP_0441 (*secE*) | 534281 | G**A**C (T) 🡪 G**G**C (A) | Missense mutation |
| *P. putida* KT2440 E1.1 | PP_2682 (*yiaY*) | 3071601 | **C**AG (Q)🡪 **T**AG (Stop) | Nonsense mutation |
| *P. putida* KT2440 E1  *P. putida* KT2440 E1.1 | PP_2682 (*yiaY*) | 3072054 | **C**GT (A) 🡪 **T**GT (C) | Missense mutation |
| *P. putida* KT2440 E1.1 | PP_4373 (*fleQ*) | 4964766 | **A** 🡪 **ACGGCGCTTCG** | Frameshift |
| *P. putida* KT2440 E1  *P. putida* KT2440 E1.1 | PP_4373 (*fleQ*) | 4964949 | **C**AG (Q) 🡪 **T**AG (Stop) | Nonsense mutation |
| *P. putida* KT2440 E1.1 | intergenic region upstream of PP_3839 (*adhP*) | 4362872 | **C** 🡪 **T** | Predicted -10 element affected |





**Supplementary Figure 1.** Predicted promoter region upstream of *adhP* using the Softberry BPROM tool (Solovyev and Salamov, 2011). Shown is the alteration of the -10 element based on the introduced mutation (bold, red) in the evolved strain *P. putida* KT2440 E1.1 compared to the reference (*P. putida* KT2440).

**Reference**

Solovyev, V., and Salamov, A. (2011). “Automatic annotation of microbial genomes and metagenomic sequences,” in *Metagenomics and its Applications in Agriculture, Biomedicine and Environmental Studies*, ed. R. W. Li (Hauppauge, NY: Nova Science Publishers), 61–78.
